# Intraoperative Characterization of Subthalamic Nucleus-to-Cortex Evoked Potentials in Parkinson’s Disease Deep Brain Stimulation

**DOI:** 10.3389/fnhum.2021.590251

**Published:** 2021-03-11

**Authors:** Lila H. Levinson, David J. Caldwell, Jeneva A. Cronin, Brady Houston, Steve I. Perlmutter, Kurt E. Weaver, Jeffrey A. Herron, Jeffrey G. Ojemann, Andrew L. Ko

**Affiliations:** ^1^Graduate Program in Neuroscience, University of Washington, Seattle, WA, United States; ^2^Center for Neurotechnology, University of Washington, Seattle, WA, United States; ^3^Department of Bioengineering, University of Washington, Seattle, WA, United States; ^4^Department of Physiology and Biophysics, University of Washington, Seattle, WA, United States; ^5^Department of Radiology, University of Washington, Seattle, WA, United States; ^6^Department of Neurological Surgery, University of Washington, Seattle, WA, United States

**Keywords:** electrocorticography, deep brain stimulation, evoked potential, subthalamic nucleus, Parkinson’s disease, high-frequency stimulation

## Abstract

Deep brain stimulation (DBS) of the subthalamic nucleus (STN) is a clinically effective tool for treating medically refractory Parkinson’s disease (PD), but its neural mechanisms remain debated. Previous work has demonstrated that STN DBS results in evoked potentials (EPs) in the primary motor cortex (M1), suggesting that modulation of cortical physiology may be involved in its therapeutic effects. Due to technical challenges presented by high-amplitude DBS artifacts, these EPs are often measured in response to low-frequency stimulation, which is generally ineffective at PD symptom management. This study aims to characterize STN-to-cortex EPs seen during clinically relevant high-frequency STN DBS for PD. Intraoperatively, we applied STN DBS to 6 PD patients while recording electrocorticography (ECoG) from an electrode strip over the ipsilateral central sulcus. Using recently published techniques, we removed large stimulation artifacts to enable quantification of STN-to-cortex EPs. Two cortical EPs were observed – one synchronized with DBS onset and persisting during ongoing stimulation, and one immediately following DBS offset, here termed the “start” and the “end” EPs respectively. The start EP is, to our knowledge, the first long-latency cortical EP reported during ongoing high-frequency DBS. The start and end EPs differ in magnitude (*p* < 0.05) and latency (*p* < 0.001), and the end, but not the start, EP magnitude has a significant relationship (*p* < 0.001, adjusted for random effects of subject) to ongoing high gamma (80–150 Hz) power during the EP. These contrasts may suggest mechanistic or circuit differences in EP production during the two time periods. This represents a potential framework for relating DBS clinical efficacy to the effects of a variety of stimulation parameters on EPs.

## Introduction

High-frequency deep brain stimulation (DBS) in the subthalamic nucleus (STN) has been commonly used to treat symptoms of advanced Parkinson’s disease (PD) since the late 1990s (Wichmann and DeLong, 2016), but its basic mechanisms remain debated. Pathophysiology of PD at the cortical level, particularly the primary motor cortex (M1), is well-established from electroencephalography (EEG) and electrocorticography (ECoG) recordings of PD patients and includes high-amplitude beta oscillations (Mallet et al., 2008; Crowell et al., 2012) and tight phase-amplitude coupling between beta and gamma frequencies (De Hemptinne et al., 2013). Some studies have observed reduction of these pathological oscillations with clinically effective DBS (Kühn et al., 2008; De Hemptinne et al., 2015), suggesting that such abnormal activity is suppressed by STN stimulation. However, our understanding of basal ganglia-cortical interactions, their role in PD, and how they are altered by DBS is limited, and these observations have not yet contributed significantly to clinical treatment (Devergnas and Wichmann, 2011).

The structural and functional circuits connecting the basal ganglia and the cortex are classically grouped into three pathways – the direct, indirect, and hyperdirect pathways. Of these, STN DBS may directly modulate the latter two (Wichmann and DeLong, 2016). The indirect pathway is a cortico-basal ganglia-thalamo-cortical loop that connects the primary input structure of the basal ganglia, the striatum, to the primary output structure, the internal segment of the globus pallidus (GPi), via the external segment of the globus pallidus (GPe) and the STN. From the GPi, the pathway then projects through motor areas of the thalamus to feed back on the motor cortex (Wichmann and DeLong, 2016). The hyperdirect pathway consists of fibers descending from motor cortical areas directly to the STN (Monakow et al., 1978; Miocinovic et al., 2018).

The ascending portions of these circuits are implicated in both the pathophysiology of PD and the therapeutic efficacy of DBS. Many hypotheses exist as to how high-frequency stimulation affects the output of the basal ganglia and how these changes improve PD symptoms (Devergnas and Wichmann, 2011). The “informational lesion” hypothesis posits that DBS activates outgoing axons of the STN, thus preventing the transmission of pathological basal ganglia activity to the cortex without disrupting the structural connectivity (Grill et al., 2004). The “selective filter” hypothesis suggests a more limited disruption that leaves some functional information transmission between STN and cortex intact while specifically blocking high amplitude, low frequency activity patterns from the basal ganglia (Agnesi et al., 2013; Zimnik et al., 2015), leading to an overall enhancement of activity in cortico-basal ganglia-thalamo-cortical loops (Fukuda et al., 2002). A third, increasingly popular hypothesis suggests that DBS disrupts pathological synchrony within the basal ganglia, thus disrupting abnormal cortical oscillations and phase-amplitude coupling entrained and propagated through cortico-basal ganglia-thalamo-cortical loops (De Hemptinne et al., 2015; Wilson and Moehlis, 2015). Further exploration of the functional impacts of STN stimulation on cortical physiology could help elucidate subtle differences between these theories.

An extensive literature has explored evoked cortical activity (e.g., EEG, ECoG) in response to single stimulation pulses in the STN (Devergnas and Wichmann, 2011). Both short- and long-latency evoked potentials (EPs) are observed at the cortex after single stimulation pulses in the STN and GPi (Devergnas and Wichmann, 2011). The short-latency (∼2–10 ms) EPs elicited by STN stimulation are temporally consistent with antidromic activation of the hyperdirect pathway, implying this circuit may be activated and/or modulated by DBS (Miocinovic et al., 2018). Longer latency (18–25 ms or longer) EPs are thought to reflect multisynaptic, orthodromic transmission through the indirect pathway (Ashby et al., 1999; Devergnas and Wichmann, 2011). While the exact significance of these EPs remains debated, it is thought that they reflect changes in cortical excitability in response to STN stimulation (Ashby et al., 1999).

One limitation of these subcortical-to-cortical EP studies is that they typically look at responses to single DBS pulses, delivered at a low frequency (typically 5–30Hz) to allow for long-latency responses uninterrupted by further stimulation pulses, which would also introduce stimulation artifacts that could obscure cortical signals (Devergnas and Wichmann, 2011). However, low frequency stimulation of STN is typically ineffective at treating symptoms of PD (Wichmann and DeLong, 2016). To better understand how therapeutic DBS impacts cortical activity, a characterization of cortical evoked activity in response to high-frequency (>100 Hz) stimulation is necessary.

This study begins to address this gap in our understanding of the functional subcortical-cortical interactions at play in high-frequency STN DBS. We combine ECoG in an intraoperative setting and a recently published artifact removal method that *post hoc* subtracts artifacts from recordings (Caldwell et al., 2020) to reveal physiological signals during ongoing DBS. We then examine cortical EPs during and immediately after trains of high-frequency stimulation that resemble clinical DBS protocols.

## Materials and Methods

### Subjects

Six subjects (6 M, ages 63–77) undergoing clinical STN implantations for DBS underwent additional, temporary placement of unilateral or bilateral subdural ECoG strips and intraoperative DBS for research purposes. All research methods were conducted in accordance with a University of Washington Institutional Review Board-approved protocol with informed consent obtained from participants. Of the 6 patients, 4 received bilateral DBS implants, 1 received only a right implant, and 1 received only a left implant. The ECoG strip was placed ipsilaterally to the electrode used for stimulation (4 left, 2 right). One patient (Subject 4) was bilaterally implanted with ECoG strips, but only the strip ipsilateral to stimulation (right) was analyzed. Subject demographics, implant information, and medications are summarized in Table 1.

**TABLE 1:** Subject demographics.

	Age	Sex	Preoperative PD medication	Disease duration	Implant hemisphere	Recording hemisphere
Subject 1	77	M	Carbidopa-levodopa	11 years	Bilateral	L
Subject 2	76	M	Carbidopa-Levodopa; Entacapone	20 years	L	L
Subject 3	69	M	Pramipexole; Carbidopa-Levodopa; Rasagiline	5 years	R	R
Subject 4	71	M	Carbidopa-Levodopa; Rasagiline; Ropinirole	8 years	Bilateral	R
Subject 5	63	M	Carbidopa-Levodopa	35 years	Bilateral	L
Subject 6	78	M	Carbidopa-Levodopa	5 years	Bilateral	L

For several reasons, we did not seek to directly examine data in relation to the therapeutic effects of DBS for individual subjects due to limited and/or inconsistent clinical follow-up data: (1) Not all patients underwent DBS programming at the University of Washington, making it difficult to obtain accurate records with extensive mapping of clinical response, (2) Patients that we do have records for were often assessed using monopolar or novel stimulation configurations, rather than the bipolar configuration used here, and (3) In at least one subject, the clinical team re-positioned the DBS electrodes after the research team collected data.

### Clinical Procedures for DBS and ECoG Placement

**FIGURE 1:**
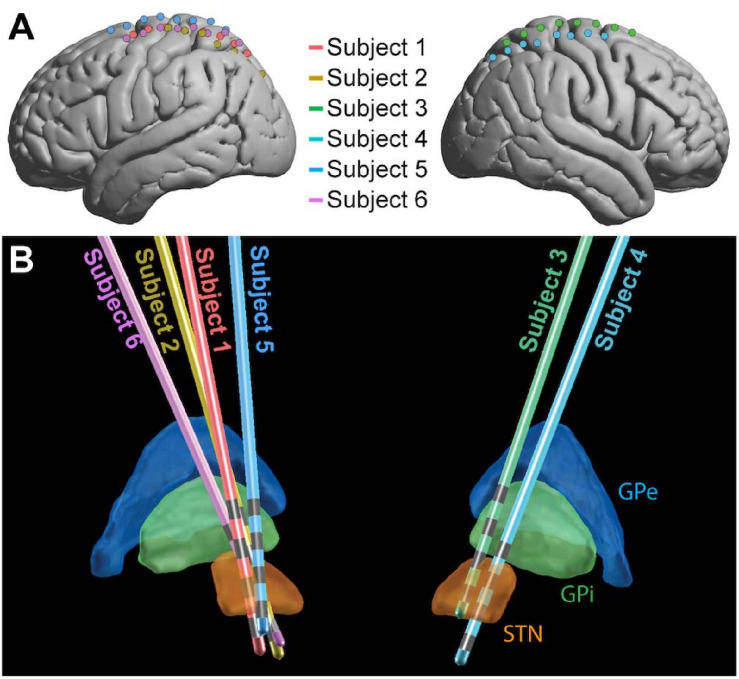
Electrode placement. ECoG **(A)** and DBS **(B)** electrode locations shown for the 6 subjects in MNI space. For ECoG strips, electrode 1 was the most posterior and electrode 8 was the most anterior. The 4 gray bands on each DBS contact **(B)** represent the contacts, with electrode 0 the deepest and electrode 3 the most superficial (Medtronic naming conventions). GPe, globus pallidus external segment; GPi, globus pallidus internal segment; STN, subthalamic nucleus.

All patients were under total intravenous anesthesia as well as PD medication (see Table 1) for the duration of the implantation and research protocols. Once in the operating room and deeply anesthetized, patients’ heads were fixed at three points using a skull clamp and long DORO Transitional Member Radiolucent headrest system (Pro Med Instruments GmbH). After affixing bone fiducials (Medtronic Inc.) to the skull, a pre-implant CT scan was obtained (see below) and registered to a pre-operative MRI using FrameLink (Medtronic Inc.). The co-registered imaging was used to form a surgical approach plan to the STN (unilaterally or bilaterally, as described above). A Stimloc burr hole cover (Medtronic Inc.) was secured to the skull and the dura mater was opened widely in a cruciate fashion over the hemisphere(s) where the ECoG strip(s) was to be placed. An eight contact macro-scale ECoG strip (2.3 mm diameter exposed surface per electrode, 1 cm inter-electrode spacing, Ad-Tec Medical Instruments Corp.) was slipped underneath the dura posteriorly, parallel to the midline, so as to approximately cross the hand/upper extremity region of primary motor cortex. A Nex-Frame frameless stereotactic system (Medtronic Inc.) was then positioned and DBS lead (1.5 mm inter-contact spacing, Medronic Inc., model 3387) placement continued as previously described (Herron et al., 2017). A second, post-implant CT was then acquired in order to confirm the position of the DBS lead. This CT was subsequently used to localize surface ECoG electrodes. For one subject (Subject 2), the clinical team repositioned the DBS lead three times in response to imaging examination, but only recordings obtained with the original implant position were analyzed. See Figure 1 for positions of ECoG (Figure 1A) and DBS (Figure 1B) electrodes for all subjects.

### Intraoperative Stimulation and Recording

All stimulation and recording for research purposes was performed with a Tucker David Technologies (TDT, Alachua, Florida, United States) acquisition system. A TDT IZ2H-16 stimulator with LZ48-400 battery pack was used to stimulate through DBS electrodes, and both STN and cortical electrodes were recorded using a System 3 RZ5D and PZ5 Neurodigitizer. A scalp EEG electrode was used as a reference for all recordings. No stimulation parameters used in this study exceeded a charge density of 60 μC/cm^2^/phase to ensure patient safety and avoid tissue damage (Cogan et al., 2016).

**FIGURE 2:**
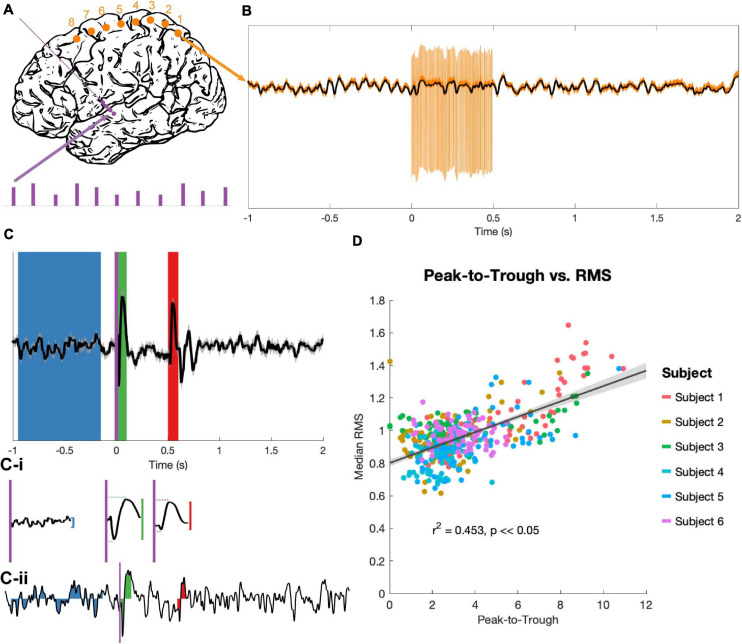
Stimulation and evoked potential measurement. **(A)** Monophasic stimulation was delivered in a bipolar configuration to DBS electrodes (purple dots). Stimulation occurred in 5 s bursts at 4 amplitudes (purple bars), though only EPs evoked by 3 V stimuli were analyzed. Signals were recorded at cortical electrodes (orange dots). **(B)** Raw trial (orange) shows stimulation artifacts, which were removed by an unsupervised dictionary-based learning algorithm (black). **(C)** The average of 30 trials (top trace, ±SEM, z-scored) was used to identify EPs. A long baseline period (blue) prior to stimulation onset (purple vertical line) and the 100 ms windows immediately after stimulation onset (t = 0 s, green) and offset (t = 0.5 s, red) were the regions of interest. The peak-to-trough amplitude was computed for each period (vertical red and green lines), as well as the latency to peak and trough components for the two EPs (horizontal red and green dashed lines, **C-i**). The RMS amplitude was extracted for these time periods for each z-scored individual trial (example trial shown in **C-ii**). **(D)** The peak-to-trough of the average trace and the median RMS of all trials (separate medians for the start and end EPs) were highly correlated across subjects in a non-parametric test (generalized linear model in black with confidence intervals in gray, r^2^ and p from Spearman correlation).

The stimulation protocol delivered a series of high-frequency stimulation trains, each of 180 (n = 4) or 185 (n = 2) Hz. Each train was 0.5 s in duration with an inter-train interval of 2.5 s (Figure 2A). Stimulation pulses were monophasic and delivered in bipolar configurations between contacts on the DBS electrodes, with pulse widths of 60 μs. 60 stimulation bursts were delivered in blocks to each consecutive bipolar configuration on the DBS lead (0–1, 1–2, 2–3), with trains divided evenly among 4 voltage levels (determined individually for each subject based on the trained clinical team’s [AK] recommendations). Each DBS electrode pair was tested in both possible bipolar configurations (i.e., anodic first and cathodic first), for a total of 360 bursts per subject. Only the 90 of these bursts delivered at 3 V (the highest stimulation voltage that all subjects had in common) were considered for analysis, yielding 30 bursts per DBS electrode pair. While DBS stimulation was delivered, recordings were obtained from the 8 cortical strip electrodes at 48 kHz.

### Imaging and Electrode Localization

Preoperative clinical Magnetic Resonance Imaging (MRI) and intraoperative Computerized Tomography (CT) were used for electrode localization and anatomical computations. A Philips 3T Achieva scanner with a standard 8 channel SENSE head coil was used to acquire high-resolution 3D T1 magnetization prepared rapid gradient echo (MRPAGE) sequence (repetition time (TR)/echo time (TE)/flip angle: 4.17/51/8°). Slice thickness was 0.750 mm, and the scan included 640 × 640 FOV matrix with 214 overlapping slices, resulting in in-plane resolution of 0.4 × 0.4 mm^3^. Intraoperative CT scans were acquired on a CereTom scanner (NeuroLogica Inc.), resulting in a 512 × 512 × 88 matrix and an in-plane resolution of 0.5 × 0.5 mm with 1.25 mm slice thickness.

The MRI and CT were co-registered using a standard affine transform in Freesurfer (http://surfer.nmr.mgh.harvard.edu/) and surface electrodes were manually identified. The electrode coordinates were then transformed into 152MNI space (1 × 1 mm). Brodmann areas were determined manually using an MNI-based atlas.

LEAD-DBS (Horn and Kühn, 2015) was used to localize DBS electrodes on co-registered MRI and CT scans and project them into MNI space. The DISTAL atlas (Ewert et al., 2018) was used in LEAD-DBS to visualize the location of electrodes relative to the thalamus and STN (Figure 1).

### Data Pre-processing

For EP analysis, minimal pre-processing was used in an attempt to preserve the shape and latency of the complex, multiphasic responses. No re-referencing was performed, as many (if not all) cortical electrodes in each subject exhibited simultaneous EPs of different sizes but similar shapes, so bipolar or common average referencing would have reversed the polarities of some of these EPs. EPs were averaged over trials during the periods of interest.

The collected time-series data were first segmented into 2.5 s epochs, each containing 1 s of rest, 0.5 s of stimulation, and another 1.5 s of rest. Epochs were then run through an unsupervised, dictionary-based artifact rejection pipeline as previously described (Caldwell et al., 2020) (Figure 2B). Briefly, this clusters each ECoG channel’s artifacts based on shape to create a dictionary, matches each individual artifact to its closest dictionary entry, and subtracts a scaled version of this template from the trace to flatten the artifact and approximate the underlying signal. Residual artifact (which was minimal) and additional high-amplitude spike-like noise (not uncommon in the intra-operative setting) lasting less than 0.5 ms were removed and the resulting gaps were linearly interpolated. Each trial was then visually examined, and trials with remaining high-amplitude noise were removed. An average of 1.67/90 trials were removed per subject.

After artifact removal, time-series epochs were lowpass filtered at 200 Hz using a fourth order Butterworth filter and down-sampled by a factor of 8, for an ultimate sampling rate of approximately 6 kHz. 60 Hz line noise and harmonics were removed with fourth order Butterworth notch filters. Finally, the data were baseline corrected by subtracting the mean voltage of a 0.5 s period before the start of each stimulation burst.

### Evoked Potential Analysis

90 stimulation trains delivered at 3 V were processed for each subject, then grouped by bipolar stimulation pair. Average EPs from the 30 traces for each recording pair and stimulation condition were calculated (for example, see Figure 2C). The 100 ms period immediately following stimulation onset (t = 0 to 0.1 s) and the 100 ms period immediately following stimulation offset (t = 0.5 to 0.6 s) were extracted as the “start” and “end” EP windows respectively. For statistical contrast, an 800 ms period prior to burst onset (t = −0.95 to −0.15 s) was defined as a baseline. A z-transform over the entire EP period was used to standardize amplitudes across subjects, then the largest amplitude difference between a consecutive peak-to-trough or trough-to-peak pair was taken (“peak-to-trough measure,” Figure 2C-i). The latencies between the start of the EP period (stimulation onset or offset) and the positive and negative peaks were also noted.

Individual trials were too variable to get a reliable peak-to-trough measure, so the root mean square (RMS) of the EP was used to quantify deflection from the zero for individual trials (Prime et al., 2017; Shimada et al., 2017). RMS values of the z-scored trials were computed for the three time windows identified above (Figure 2C-ii). Because RMS is an average-based measure, having a longer baseline period did not inflate the values as compared to the EP periods, and a longer baseline allowed for a more stable estimate of “baseline” activity despite high trial-to-trial variability.

### Spectral Analysis

Low gamma (30–80 Hz) and high gamma (80–150 Hz) power series were also constructed for each artifact-free trial, following the pre-processing steps described above. Fourth order Butterworth bandpass filters for the low and high gamma frequencies were applied to individual trials, then the square of the analytical amplitude from the Hilbert transform was taken as power time series. These were then baseline normalized to a period 0.5 to 0.1 s prior to stimulation onset using a z-transform (the median rather than the mean was used for the average because of the unstable baseline) to correct 1/f scaling. Using this baseline rather than the 800 ms baseline used in EP analysis allowed us to better avoid edge artifacts, leaving a 500 ms buffer on the front end of each trial. The median of the gamma-filtered series was taken over each set of trials to construct a single power series for each stimulation-recording electrode pair.

### Statistics

To identify non-zero EPs, the distributions of RMS measures for each trial of the EP period were compared to the RMS distribution of the corresponding trial’s baseline using a non-parametric, paired (signed rank) test. EPs that differed significantly (*p* < 0.05, FDR-corrected) from the baseline period were counted as “significant EPs” The median RMS of all trials for each significant EP, along with the latency values recorded from the average trace, was contrasted between the two EP periods using a non-parametric, unpaired (Wilcoxon rank-sum) test (significance cutoff of *p* < 0.05, FDR corrected).

RMS and latency values were then split by the Brodmann area (BA) of the corresponding cortical electrode. Only BAs with consistent coverage among subjects (1/2/3, 4, 6, and 7) were included in these analyses, although values for all other BAs (19, 39, 40) were pooled together and shown for transparency. Evoked activity for all recording electrodes and stimulation conditions, not just significant EPs, were included in this analysis. The start and end EP values for each metric were compared within each BA using Wilcoxon rank-sum test, and then the effect of BA on each metric within the start and end EPs was determined using a non-parametric, one-way analysis of variance (Kruskal-Wallis test). For metrics and EPs for which a significant (*p* < 0.05, FDR-corrected) effect was detected, *post hoc* testing (using MATLAB’s *multcompare* command) was performed to reveal significant differences between individual BAs.

A mixed linear model was used to assess the relationships between high and low gamma and EP magnitude (RMS), adjusting the intercept and slope for random effects of subject on EP and spectral data. From the high and low gamma power series for each stimulation-recording electrode pair (3 stimulation electrodes and 8 recording electrodes for 24 pairs per subject, see Section 2.7), the median power during the start (0–0.1 s after stimulation onset) and end (0.5–0.6 s after stimulation onset) EP periods was extracted. This was regressed against the median RMS over all trials for each stimulation-recording electrode pair. We adjusted our model to control for random effects of subject on EP RMS and gamma power.

## Results

After removing stimulation artifacts from cortical recordings obtained during DBS, we quantified EPs during ongoing stimulation and compared them to EPs following stimulation offset.

### Measures of EPs

The peak-to-trough measurement of the average EP (computed from the average of 30 trials) and the RMS measurement of the same EP (the median of the RMS measures over the same 30 trials) correlated tightly (r^2^ = 0.453, *p* < 0.05; Figure 2D). Because the RMS provides a magnitude distribution rather than a single magnitude value for each EP, it was used as the primary measure of EP size for all statistics.

### Characterizing Start and End EPs

**FIGURE 3:**
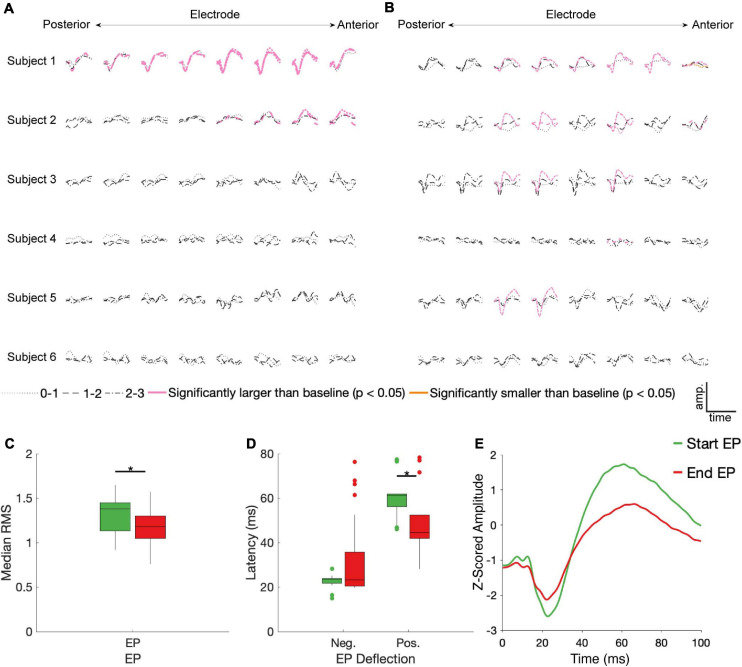
STN DBS evokes start and end EPs. Z-scored start **(A)** and end **(B)** EPs (average of 30 trials) are shown for each electrode (columns, contacts were in different BAs for each subject – see Figure 1), subject (rows), and DBS stimulation electrode pair (line type). EPs in pink had a statistically significantly larger magnitude than baseline deviations (*p* < 0.05, FDR corrected by subject). The one trace in yellow was also statistically different than baseline, but it had a lower magnitude. Median RMS **(C)** and latencies to negative and positive deflections on the average traces **(D)** were compared between the start and end EPs; significant (*p* < 0.05, FDR corrected) differences indicated by stars. **(E)** The average of all significant start (green) and end (red) EPs.

23/90 and 21/90 stimulation conditions produced EPs during the first 100 ms of stimulation and immediately following stimulation offset, respectively, that differed significantly from baseline (Figures 3A,B). Start EPs were observed in only two subjects, with the majority (∼75%) seen in Subject 1. Conversely, at least one significant end EP was observed in 5/6 subjects. Most EPs (start and end) had a characteristic biphasic shape with a narrow negative deflection followed by a longer positive deflection (Figure 3E). We noted similar features in other stimulus-triggered averages during the start and end windows that did not meet the statistical criteria. For example, Subject 3, channel 7, stimulation condition 2-3 and Subject 5, channel 6, stimulation condition 1-2 may have small start and end EPs respectively.

No discernable pattern was seen connecting the location of the stimulated electrodes to any features of the EPs. Of the 12 cortical electrodes with significant start EPs, 8 had significant EPs in more than one stimulation configuration, while 11/16 cortical electrodes had significant end EPs in multiple conditions. 9 cortical electrode-STN stimulation site pairs had significant start *and* end EPs.

Among the significant EPs, the magnitude (as measured with RMS) of start EPs were significantly larger than that of end EPs (median start RMS = 1.38, median end RMS = 1.18, *p* = 0.018; Figure 3C). Additionally, relative to stimulus onset and offset respectively, the positive deflection of start EPs occurred significantly later than that of end EPs (median start latency = 61.19 ms, median end latency = 44.56 ms, *p* = 2.32e-4; Figure 3D). No significant difference in negative deflection latency was observed between EPs, although there was a trend toward the start EP having a shorter latency to the negative deflection (median start latency = 23.51 ms, median end latency = 23.27 ms, *p* = 0.055 Figure 3D). This was likely driven by the much greater degree of variability in this latency for the end EP.

### EPs by Brodmann Area

**FIGURE 4:**
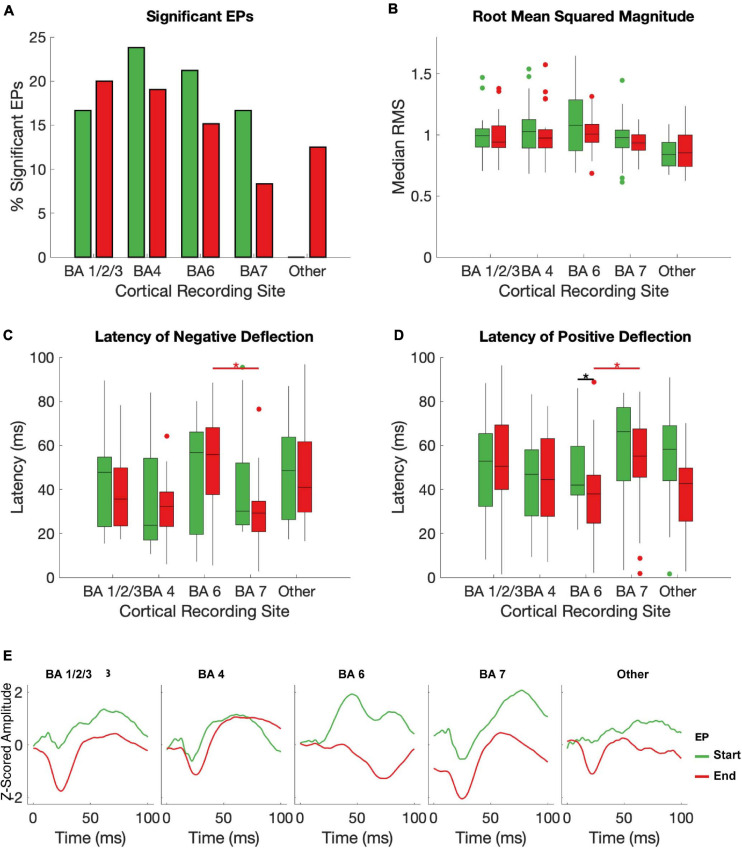
Effects of brodman Area on EPs. The median RMS **(A)**, percent of EPs that differed significantly from baseline amplitudes **(B)**, latency to negative deflection **(C)**, and latency to positive deflection **(D)** were compared between start and end EPs with recording electrodes grouped by Brodman area across subjects. Brodmann areas with inconsistent coverage across subjects were pooled and shown for comparison but not included in statistical analysis. Black stars indicate significant (*p* < 0.05, FDR corrected) differences between start and end EP measures within each BA. Red stars indicate significant (*p* ≤ 0.05, FDR corrected) *post hoc* comparisons between end EPs in different regions after a Kruskal-Wallis test revealed dependence of both latency measures on BA. The mean of all start (green) and end (red) EPs for each electrode in each BA are shown in **(E)**.

BA1/2/3 (primary sensory cortex, S1), BA4 (primary motor cortex, M1), and BA6 (premotor and supplementary motor areas, PMA/SMA) all had a >10% chance of producing a significant start and/or an end EP in response to STN stimulation (Figure 4A). All subjects had one or more electrodes on each of these BAs. BA7 (visuo-motor coordination area) also had a relatively high likelihood of producing a start EP and a non-zero likelihood of producing an end EP. In other BAs represented (BA19, associative visual area; BA 39, angular gyrus; and BA40, supramarginal gyrus – areas not primarily associated with sensorimotor function), no start EPs and few end EPs were elicited that differed significantly from baseline. The averages of significant EPs (Figure 4E) was generally similar between BAs, following the overall patterns seen in Figure 3E. The exception was in BA6, where the characteristic biphasic EP shape was less clear.

Within individual BAs, RMS and deflection latencies of start and end EP periods were not statistically distinct, despite the overall differences seen in the pooled EPs, except in one case – in BA 7, the positive deflection of the start EP occurred significantly later than that of the end EP (median start latency = 42.02 ms, median end latency = 38.01 ms, *p* = 0.0034; Figures 4B–D). Across BAs, there was a significant effect on both the positive (*p* = 6.4204e-5) and negative (p = 0.0043) latency of the end EP only. *Post hoc* testing revealed that this was driven by statistical differences between BAs 6 (median negative deflection latency = 55.87 ms, median positive deflection latency = 38.01 ms) and 7 (median negative deflection latency = 29.25 ms, median positive deflection latency = 55.13 ms) in the case of both the negative (*p* = 0.0026) and positive (*p* = 2.3087e-5) deflections. These data are summarized in Table 2.

**TABLE 2:** Start and end EPs by BA.

		BA 1/2/3	BA 4	BA 6	BA 7	Other	*p*-value (Effect of BA, *post hoc* tests for significant effects)
RMS	Start EP	0.993	1.027	1.054	0.976	0.838	0.348
	End EP	0.941	0.974	0.978	0.934	0.854	0.327
	*p*-value (Start vs. End)	0.309	0.715	0.159	0.414	−	−
Negative deflection latency	Start EP (ms)	47.759	23.757	56.689	30.147	48.579	0.047
	End EP (ms)	35.635	32.276	55.951	29.245	40.960	0.004* BA 1/2/3-4: 0.962 BA 1/2/3-6: 0.0362 BA 1/2/3-7: 0.268 BA 4-6: 0.018 BA 4-7: 0.675 BA 6-7: 2.31 × 10^–5^
	*p*-value (Start vs. End)	0.805	0.903	0.016	0.096	−	−
Positive deflection latency	Start EP (ms)	52.838	46.858	42.844	66.273	58.245	0.312
	End EP (ms)	50.545	44.564	38.093	55.132	42.680	6.42 × 10^–6^ BA 1/2/3-4: 0.896 BA 1/2/3-6: 0.0485 BA 1/2/3-7: 0.849 BA 4-6: 0.356 BA 4-7: 0.463 BA 6-7: 0.0026*
	*p*-value (Start vs. End)	0.159	0.715	0.411	0.003*	−	−

*Median RMS (Figure 4B) and median latency to negative (Figure 4C) and positive (Figure 4D) deflections by Brodmann area (BA). For each measure and BA, the *p*-value from a sign rank test for difference between start and end EP is shown. Additionally, the results of a Kruskal-Wallis test for effects of BA on EPs are given for all measures and for both the start and end EPs. For tests with significant (*p* < 0.05, FDR corrected) values, the *p*-values obtained from *post hoc* testing is listed for all pairs of BAs.*

### High and Low Gamma Power During EPs

**FIGURE 5:**
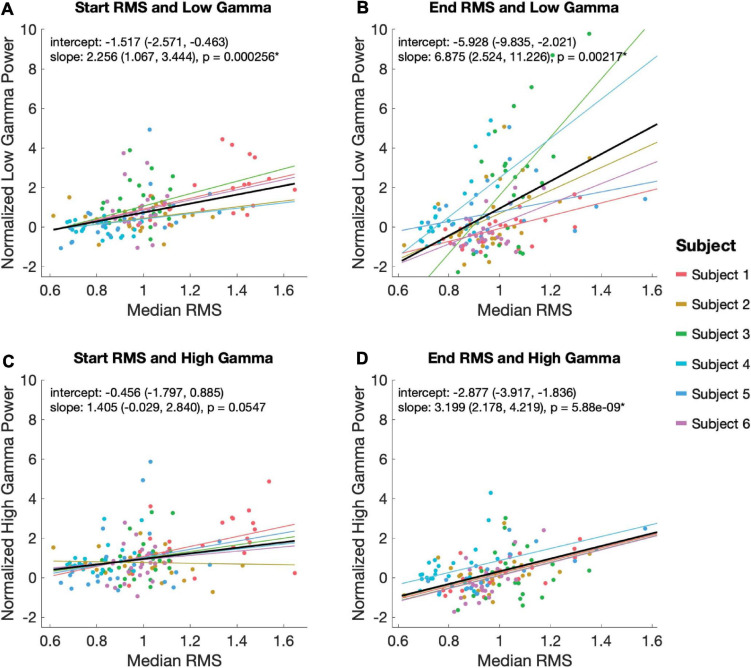
Relationship between EP magnitude and gamma power. A mixed linear model was used to assess the relationship between EP magnitude low (30–80 Hz; **A,B**) and high (80–130 Hz; **C,D**) gamma power during the start **(A,C)** and end **(B,D)** EP intervals. The model controlled for random effects of subject, adjusting for intercept and slope. Each plot shows the model’s predicted fits for each subject as well as the overall model in black. Overall model and slope are reported along with 95% confidence intervals.

Using a linear mixed effects model with subject as a random variable, we tested the relationship between low and high gamma power with RMS during the start and end EP windows. Statistically significant relationships were observed between low gamma power during the 100 ms EP windows and magnitude of both the start (slope = 2.56, *p* = 2.56 × 10^–4^) and end (slope = 6.875, *p* = 0.00217) EPs (Figures 5A,B). High gamma power during the same windows correlated significantly with the magnitude of the end (slope = 3.199, *p* = 5.88 × 10^–9^), but not the start (slope = 1.405, *p* = 0.0547) EP (Figures 5C,D).

## Discussion

With acute, intraoperative subdural ECoG implants, we measured evoked potentials and power spectra at a number of cortical sites in response to high-frequency stimulation of the STN in PD patients. After reliable removal of the stimulation artifact (Caldwell et al., 2020), we observe two distinct responses that resemble canonical subcortico-cortical EPs – one during the first 100ms of a high-frequency, 500 ms stimulation train (“start EP”) and one during the 100ms immediately following the offset of these trains (“end EP”). This provides additional characterization of EPs in cortex during ongoing, continuous and clinically relevant DBS. Within the framework of classic informational lesioning or transmitter depletion theories of STN DBS, the novel end EP may be indicative of a “rebound” in cortical activity after high-frequency DBS is turned off.

Responses at the cortex in response to high frequency STN DBS have previously been reported in EEG (Baker et al., 2002), but have not been quantified due to lack of sufficient artifact removal techniques. Although Baker and colleagues noted slow wave oscillations during stimulation artifact of 100ms of stimulation, their primary focus was on slower EPs that occurred after termination of the stimulation artifact (Baker et al., 2002). Our observed deflections during ongoing stimulation are more pronounced than previously reported. This discrepancy potentially may be due to a closer proximity to the dipole source and resulting higher fidelity of ECoG recordings with respect to EEG. It is unclear whether the slow oscillations (∼140–230 ms after stimulation onset, or ∼40–130 ms after stimulation offset) in the EEG is similar to the end EPs described here. Although there is some overlap in the time window, the first component of the observed end EP is still faster than the earliest component reported by Baker et al. More work is necessary, including varying the length of stimulation period, to elucidate these discrepancies.

The start and end EPs are grossly similar in shape (i.e., wave morphology) and are consistent with previously reported subcortico-cortico EPs (Hartmann et al., 2018; Miocinovic et al., 2018). We do, however, note some quantitative differences between the two EPs. The start EPs tend to be larger and longer than the end EPs. Additionally, start EP magnitude correlates with both high and low gamma power, while end EP magnitude correlates only with low gamma power. These differences may be attributed to several unique and/or overlapping possibilities, including, (1) residual DBS artifacts surviving our artifact removal process, (2) evoked responses within distinct pathways and mechanisms, or (3) modulation of EP elements from ongoing high-frequency DBS.

We further note that, consistent with previous DBS studies, medium- to long-latency EPs are seen primarily in cortical areas corresponding to sensorimotor function and integration, consistent with the signals traveling through known cortico-basal ganglia-thalamo-cortical loops of the indirect pathway. The site of stimulation in relation to the STN did not have a significant effect on EP magnitude or on the likelihood of producing a statistically significant (larger than baseline) EP, which may indicate that current spread within any given bipolar electrode configuration may play a role in the transmission of these signals from the STN to the relevant tracts.

### Start vs. End EPs

The EPs we observe immediately following stimulation onset and offset resemble previously described STN-to-cortex EPs in shape and latency – a negative deflection around 20–30 ms and a positive deflection around 40–60 ms following stimulation onset or offset (Hartmann et al., 2018; Miocinovic et al., 2018). These canonical EPs are observed in ECoG (Miocinovic et al., 2018), magnetoencephalography (Hartmann et al., 2018), and electroencephalography (Walker et al., 2012). Their timing is consistent with orthodromic, multisynaptic transmission (Walker et al., 2012; Hartmann et al., 2018). The circuits through with this occurs are, as of yet, not fully understood, but modeling and experimental evidence have suggested that high-frequency stimulation activates STN efferents to the GPi and/or directly activates pallido-thalamic fibers (McIntyre et al., 2004; Miocinovic et al., 2006), indicating propagation through the indirect pathway.

Our ability to generalize the findings about start EPs is limited because the majority of start EPs (∼75%) were seen in a single subject (Subject 1). Classic EP-like waveforms that did not achieve statistical differences in magnitude over baseline periods were, however, seen in other subjects during both EP periods. Larger sample sizes and ranges of stimulation amplitude and quantification of EP thresholds are clearly needed to determine the relative prevalence of start and end EPs and determine if large, consistent start EPs like those seen in Subject 1 are associated with electrode position, disease process, or any other factors.

Interestingly, we do not see any evidence for short-latency EPs, like those previously reported (Miocinovic et al., 2018), after individual stimulation pulses within a stimulation train. These are generally attributed to antidromic activation of the hyperdirect pathway and have expected latencies of 2–10 ms. With the high frequency (180–185 Hz) stimulation used in this study, responses longer than ∼5 ms would be obscured by the next stimulation pulse in the train, so responses of the expected length may not be visible in our data. More work will need to be done to see if the long-latency start and end EPs have an impact on short-latency EPs following individual stimulation pulses.

### Effect of Broadmann Area on EPs

Overall, we observed a greater likelihood of seeing an EP with a significantly larger magnitude than baseline in Brodmann Areas (BAs) associated with sensorimotor function than in any other BAs. This is consistent with the hypothesis that EPs are propagated through motor cortico-basal ganglia-thalamo-cortical loops, of which the STN is a part (McIntyre et al., 2004; Miocinovic et al., 2006; Devergnas and Wichmann, 2011).

Magnitude of EPs did not vary significantly with the BAs from which the EPs were recorded, nor did latency to either peak of the start EP. There was a significant effect of BA on end EP peak latencies, which was revealed by *post hoc* testing to be a result of differences between BA6 and BA7. The EPs observed in BA6 are less like the consistent biphasic EPs seen in S1, M1, and BA7, and the start and end EPs have approximately opposite polarities – the positive deflection comes before the negative deflection in the end EP of BA6, whereas negative comes before positive in all other EPs over sensorimotor areas. This polarity shift is responsible for the significant differences seen between start and end latencies within BA6, and likely also contributes to the overall effect of BA on EP latency without greatly impacting EP magnitude.

### Relationship Between EP Magnitude and High and Low Gamma Power

In addition to measuring time-locked evoked potentials during the periods immediately following stimulation onset and offset, we also extracted high-frequency power responses. Average low gamma (30–80 Hz) power during both time windows correlated significantly with the magnitude of EPs seen in the same windows, but a similar correlation with high gamma (80–150 Hz) power was only seen for the end EP. The functional distinction of low gamma activity in the cortex is debated. Some reports have associated low gamma power with cognitive function and stimulus dependence (Başar, 2013), while others have found that the lower end of this frequency range more closely resembles canonical beta oscillations in movement-related amplitude modulation (Unterweger et al., 2020). High gamma power is known to correlate tightly to firing rates of local neural populations and is therefore often interpreted as a measure of local activity (Ray et al., 2008). The findings here suggest that the impact of high-frequency electrical stimulation of STN may be associated not only with EP production, but also with higher stimulus-dependent activity – in this case, some variant of motor processing. However, a higher rate of cortical neuronal activity seems unique (with respect to the selected windows used in our analysis) to the period at the end of ongoing high-frequency stimulation. The mechanisms giving rise to observed distinctions in RMS-high gamma power associations between analysis windows are unclear. Among other possibilities, it is conceivable that this association is an effect of DBS entrainment or evoked processing within local circuitry. Additionally, there is a trend in the data toward a relationship between start EP RMS and high gamma power that might reach statistical significance if we had additional trials.

### Study Limitations

Many aspects of this study limit our ability to firmly draw conclusions. One limitation is that all subjects were anesthetized for the duration of this study, which has been found to change cortical oscillation patterns and lower evoked potential thresholds. Additionally, due to restricted time with each subject in the operating room, the number of trials we were able to run for each subject was highly limited. Our EPs are the average of only 30 trials, but we expect that we would see similar results but greater consistency if more trials were added, enhancing our statistical power. ECoG EPs are regularly characterized clinically with only 10s of trials, and studies have been published using as few as 20 trials per EP (e.g., Matsumoto et al., 2007; Keller et al., 2014).

As noted previously, our inability to relate our data to clinical follow-up is a significant limitation. Further behavioral work must be performed before the clinical relevance of these findings is established. Our predictions of potential relevance to clinicians are outlined below but are entirely speculative.

### Potential Clinical Relevance of EPs in Response to High-Frequency DBS

While DBS is often effective in treating PD, symptom relief varies from patient to patient. Personalizing a DBS treatment plan to fit an individual’s needs and best treat their symptoms is a time-consuming process for both clinicians and patients during which multiple stimulation parameters are tuned via behavioral testing. Better understanding how high frequency stimulation affects patterns of transmission between the STN and upstream cortical areas may provide insight into more efficient ways of individualizing therapies. If measurable events at the cortex during ongoing high frequency STN stimulation correlate with therapeutic efficacy of the stimulation parameters, these events could serve as a biomarker to more rapidly test a series of stimulation parameters without exhaustive behavioral testing. Doing this intraoperatively or postoperatively would narrow the parameter space for behavioral testing.

Additionally, better understanding basal ganglia-to-cortex functional connections could contribute to engineering new DBS devices, such as devices that pair cortical recording and/or stimulation with traditional STN stimulation to try to maximize efficacy in all patients. In order to determine how cortical and subcortical devices could work synergistically, we need a quantitative metric of their functional connectivity to test how the neural circuits respond to different types of stimulation. Previous high-frequency STN DBS efforts for example has examined EMGs modulation (Weaver et al., 2020). The EPs we observe during high-frequency DBS may represent the basis for this kind of metric.

## Conclusion

We demonstrate the existence of two cortical evoked potentials in response to high-frequency stimulation of the STN similar to that used clinically for DBS to treat PD. One EP occurs immediately after stimulation starts and is, to our knowledge, the first long-latency cortical EP reported during ongoing stimulation. A lack of effective artifact removal methods has made measuring activity during continued stimulation difficult up until recently. The second EP occurs after the offset of high-frequency stimulation, and intriguingly suggests some sort of cortical “rebound” when DBS is turned off. Significant further work will be required to elucidate the mechanisms by which these EPs are produced and if and how they are related to the therapeutic efficacy of DBS. Here, we provide a foundation for that work by describing this cortical evoked activity.

## Data Availability Statement

The raw data supporting the conclusions of this article will be made available by the authors, without undue reservation.

## Ethics Statement

The studies involving human participants were reviewed and approved by University of Washington Institutional Review Board. The patients/participants provided their written informed consent to participate in this study.

## Author Contributions

DC, JC, KW, and AK conceptualized and designed the experiment. AK performed all clinical procedures. DC, JC, BH, and KW collected data. LL analyzed the data, with guidance from SP, KW, JH, JO, and AK. LL, SP, KW, JH, JO, and AK interpreted the data. LL prepared the manuscript. SP, KW, JH, JO, and AK edited the manuscript. All authors contributed to the article and approved the submitted version.

## Conflict of Interest

The authors declare that the research was conducted in the absence of any commercial or financial relationships that could be construed as a potential conflict of interest.
